# Supplementary material for: Discussing Personalized Prognosis Empowers Patients with Amyotrophic Lateral Sclerosis to Regain Control over Their Future: A Qualitative Study
Source: Brain Sci. 2021 Nov 30;11(12):1597. doi: 10.3390/brainsci11121597 (PMC8699408; doi:10.3390/brainsci11121597)
Supplement: Supplementary file 1 [file brainsci-11-01597-s001.zip › brainsci-1448670-supplementary.pdf]

brain sciences

MDPI

Supplemental Material

Table S1. COREQ checklist.

Topic	Item No.	Guide questions/Description	Answer
		Domain 1: Research team and reflexivity	
		Personal characteristics	
Interviewer/facil-itator	1	Which author/s conducted the interview or focus group?	See page 5.
Credentials	2	What were the researcher's credentials? E.g. PhD, MD	See Table S4.
Occupation	3	What was their occupation at the time of the study?	See Table S2.
Gender	4	Was the researcher male or female?	Not relevant for this study.
Experience and training	5	What experience or training did the re-searcher have?	See page 5.
		Relationship with participants	
Relationship es-tablished	6	Was a relationship established prior to study commencement?	Participants were contacted by phone prior to the interview and informed about the study. The role of the interviewers was explained to participants. Other than that the interviewers were unknown to participants. See page 5.
Participant knowledge of the interviewer	7	What did the participants know about the re-searcher? e.g., personal goals, reasons for do-ing the research	No background knowledge of the interview-ers was known to the participants, except for their role in the study. The background of the study was explained to participants. See page 5.
Interviewer char-acteristics	8	What characteristics were reported about the interviewer/facilitator? e.g., Bias, assump-tions, reasons and interests in the research topic	Bothe interviewers were not involved in pa-tient care. See page 5.
		Domain 2: Study design	
		Theoretical framework	
Methodological orientation and Theory	9	What methodological orientation was stated to underpin the study? e.g., grounded theory, discourse analysis, ethnography, phenome-nology, content analysis	See page 4.
		Participant selection	
Sampling	10	How were participants selected? e.g., purpos-ive, convenience, consecutive, snowball	See page 4.
Method of ap-proach	11	How were participants approached? e.g., face-to-face, telephone, mail, email	See page 4.
Sample size	12	How many participants were in the study?	See page 6 and Table 1; Table 2.
Non-participa-tion	13	How many people refused to participate or dropped out? Reasons?	Two patients and one caregiver, who were in-vited by the physician to participate in this study and who agreed to participate, declined at a later time to participate. Both patients de-clined because they did not have sufficient time and energy to participate in the study;

			the caregiver did not give a reason for declining to participate.
		Setting	
Setting of data collection	14	Where was the data collected? e.g., home, clinic, workplace	See Table 1.
Presence of non-participants	15	Was anyone else present besides the participants and researchers?	During the interviews no one else was present except for the participants and the interviewers.
Description of sample	*16*	What are the important characteristics of the sample? e.g., demographic data, date	See Tables 1 and 2.
		Data collection	
Interview guide	17	Were questions, prompts, guides provided by the authors? Was it pilot tested?	The interview guide was not field tested. Topic guides have been included as supplemental tables (S3 and S4).
Repeat interviews	18	Were repeat interviews carried out? If yes, how many?	No repeat interviews were conducted.
Audio/visual recording	19	Did the research use audio or visual recording to collect the data?	Yes, see page 5.
Field notes	20	Were field notes made during and/or after the interview or focus group?	Field notes were made during the interviews to support the interviewer. These were not analysed or recorded after the interview.
Duration	21	What was the duration of the inter views or focus group?	See page 6.
Data saturation	22	Was data saturation discussed?	Yes, see page 5 and 6.
Transcripts returned	23	Were transcripts returned to participants for comment and/or corrected?	Yes, see page 5.
		Domain 3: analysis and findings	
		Data analysis	
Number of data coders	24	How many data coders coded the data?	Two, see pages 5 and 6.
Description of the coding tree	25	Did authors provide a description of the coding tree?	The coding tree is available (in Dutch) at request from the corresponding author.
Derivation of themes	26	Were themes identified in advance or derived from the data?	Derived from the data, see pages 5 and 6.
Software	27	What software, if applicable, was used to manage the data?	See page 5.
Participant checking	28	Did participants provide feedback on the findings?	No.
		Reporting	
Quotations presented	29	Were participant quotations presented to illustrate the themes/findings? Was each quotation identified? e.g., participant number	Yes, see Tables 3–6.
Data and findings consistent	30	Was there consistency between the data presented and the findings?	Yes, see Tables 3–6 and Results section (pages 6–9).
Clarity of major themes	31	Were major themes clearly presented in the findings?	Yes, see pages 6–9.
Clarity of minor themes	32	Is there a description of diverse cases or discussion of minor themes?	Yes, see pages 6–9.

Table S2. Researcher credentials.

Name	Occupation
Remko M. van Eenennaam, MSc	Researcher (PhD-student)
Loulou S. Koppenol, MSc	Researcher (Master-student)
Willeke Kruithof, MD, PhD	Rehabilitation physician
Esther Kruitwagen-van Reenen, MD, PhD	Rehabilitation physician
Sotice Pieters, MD	Rehabilitation physician
Michael van Es, MD, PhD	Neurologist
Leonard H. van den Berg, MD, PhD	Neurologist
Anne Visser-Meily, MD, PhD	Rehabilitation physician
Anita Beelen, PhD	Senior researcher

Table S3. Interview Guide (with prompts) for Interviews with Patients and Their Caregivers on Discussing Personalized Prognosis in Amyotrophic Lateral Sclerosis

1. Can you tell me how the personalized prognosis was discussed with you?
   - Who took the initiative?
   - Were your information needs met?
   - Were your other needs met?
   - Was the prediction model discussed with you?
   - Were there differences between patient and caregiver experiences?
2. Can you tell me about the impact of discussing your prognosis?
   - Emotional impact
   - Hope
   - Distress
   - Trust in physician
3. What is your life expectancy?
4. How satisfied are you with how your personalized prognosis was discussed with you?
   - What could be done to improve prognostic disclosure?

Table S4. Interview Guide (with Prompts) for focus group with physicians

1. Do you always offer new patients the option to discuss their personalized prognosis?
   - Who takes the initiative?
   - How do you offer this to patients?
   - Why do patients want to know their personalized prognosis?
2. Do you experience discussion of personalized prognosis as more difficult or stressful compared to other topics?
   - Has your training prepared you sufficiently for prognostic disclosure?
   - Do you require more support?
3. What is the impact of prognostic disclosure on patients and their caregivers?
   - How do they react to their prognosis?
   - What are do's and don'ts when discussing the prognosis?
   - How do patients differ in their reaction?
   - Is there a difference between patients and caregivers?
   - Have you or the patients/caregivers returned to the topic during later consultations?
4. What are your experiences with the prediction model?
   - What are your experiences filling out the model?
   - What are your experiences with communicating the model and outcome to the patient/caregiver?
